# Sirenomelia or mermaid syndrome with a cleft lip in a Tanzanian newborn: a case report

**DOI:** 10.1186/s13256-024-04549-5

**Published:** 2024-05-06

**Authors:** Zakaria Ismail Wilfred, Ng’weina Francis Magitta

**Affiliations:** 1 Department of Internal Medicine, School of Medicine & Dentistry, University of Dodoma, Dodoma, Tanzania; 2 Department of Biochemistry & Clinical Pharmacology, Mbeya College of Health & Allied Sciences, University of Dar es Salaam, Mbeya, Tanzania

**Keywords:** Antenatal care, Congenital malformation, Embryonic caudal region, Mermaid syndrome, Sirenomelia

## Abstract

**Background:**

Sirenomelia or sirenomelia sequence, also known as mermaid syndrome, is a rare congenital anomaly involving the caudal region of the body. The syndrome is characterized by partial or complete fusion of lower extremities, renal agenesis, absent urinary tract, ambiguous external genitalia, imperforate anus, and single umbilical artery. Sirenomelia is often associated with several visceral congenital malformations, rendering it invariably incompatible with extrauterine life.

**Case presentation:**

We present the case of 22-year-old Black African woman who delivered a term newborn by caesarean section at a gestation age of 37 weeks due to obstructed labor with fetal distress. The newborn was a fresh stillbirth weighing 2100 g and had fusion of the lower extremities, a single upper limb, ambiguous genitalia, imperforate anus, and a cleft lip. The mother had made only two prenatal visits, at which she was found to be normotensive and normoglycemic. She was not screened for routine fetomaternal infections and missed supplementation for folic acid during the critical first trimester. She did not undergo any obstetric ultrasonography. The parents of the newborn were not close relatives and there was no family history of consanguinity. Further genetic testing was not performed due to lack of laboratory capacity, and post mortem examination was not permitted due to cultural taboo and restrictions relating to handling of deceased newborns.

**Conclusion:**

Sirenomelia is a rare congenital malformation with very poor prognosis. Specific interventions during pre-conception and early prenatal care are critical in the prevention of specific congenital anomalies. Early obstetric ultrasonography is invaluable for diagnosis of sirenomelia as well as counseling for possible termination of pregnancy.

## Background

In 1542, Rocheus reported sirenomelia (SML) or mermaid syndrome for the first time, and a decade later, SML was further characterized by Palfyn [1]. Moreover, in 1961, Duhamal defined SML as the most severe form of the spectrum of caudal regression syndrome (CRS) [2]. However, SML is currently considered as a separate syndrome, with defining features of the presence of a single umbilical artery and renal agenesis [2]. SML is an extremely rare multisystem congenital malformation that usually affects the development of the embryonic caudal region (ECR) [3, 4]. Typically, it is characterized by fusion of the lower limbs, renal agenesis, ambiguous external genitalia, imperforate anus, and single umbilical artery [5, 6]. The syndrome has an incidence of 1 in 100,000 births with male:female ratio of 3:1, more commonly occurring among diabetic mothers [6]. To date, there are an estimated 300 patients with SML reported worldwide, and it is invariably lethal, with only three patients reported to survive beyond 10 years of age [6, 7].

The pathogenesis of SML is not well understood. However, it is believed to occur in individuals with a genetic predisposition whose expression is heralded by poorly understood environmental triggers. The putative triggers include exposure to heavy metals, retinoic acid, teratogenic drugs, nicotine use and excessive alcohol consumption and infection with chlamydia trachomatis [8, 9]. Additional risk factors include maternal age below 20 years or above 40 years, diabetes, and other poorly characterized agents [10–12]. The overall pathogenic insult leads to the impaired perfusion of ECR as speculated by the current three hypotheses, namely: (a) aberrant embryonic viteline network or vascular steal hypothesis, (b) impaired blastogenesis during the final stages of gastrulation with associated abnormal angiogenesis, and (c) mechanical compression of ECR [13, 14]. Thus, hypoperfusion of ECR triggers ischemic responses, which culminate in impaired organogenesis underlying the malformation of ECR, a precursor for fetal urogenital tract, gastrointestinal tract, and caudal spine.

The current genomic analysis indicates that almost all of the human fetuses with SML have a normal karyotype. Aberrant signaling responsible for the embryological development of the fetal caudal body seems to be responsible for the development of SML [13]. However, to date there are no identifiable genes responsible for SML in humans. However, the only known mutation associated with congenital caudal anomalies is the mutation in the homeobox-containing gene, *HLXB9*, which is associated with Currarino syndrome, an autosomal dominant sacral agenesis characterized by pelvic malformations, anal atresia, meningomyelocele, and urogenital defects without features of SML [15]. The available evidence from mice models indicates that SML is inherited through autosomal dominant manner. Intriguingly, SML phenotype has been demonstrated in knockout mice carrying mutations at or near T locus in the *brachyury* gene and *axin1* gene, which are normally involved in the structural development of tail and caudal body. Moreover, a de novo mutation called sirenomelia (*srn*) has been shown to cause hind limb fusion in mice [16].

Recently, mice models have provided insight on the pathogenesis of SML based on the existing hypotheses of vascular steal and aberrant blastogenesis [14, 17]. For instance, experimental mice with mutation of the *bone morphogenesis protein 7* (*Bmp7*) or *Cyp26a1* genes have been identified to cause a SML phenotype in offspring, indicating their putative role in the pathogenesis of SML [9]. Firstly, in experimental mice, a decreased Bmp signaling along with a loss of twisted gastrulation (Tsg) in ECR results in the SML phenotype [18]. *Bmp7* belongs to the transforming growth factor-β (*TGF-β*) superfamily, which is key in the signaling pathway involved in the formation of the ventroposterior mesoderm during embryogenesis [19, 20]. *Tsg* encodes for a Bmp7-binding protein, which functions as a regulatory protein, specifically as an activator of the inhibitor of *Bmp7* [21]. Thus, aberrant signaling involving either *Bmp7* or *Tsg* results in the impaired formation of caudal mesoderm of the developing embryo [18]. Secondly, retinoic acid (RA), an active form of vitamin A, is involved in the regulation of the formation of vascular networks in ECR [22, 23], whose expression is tightly regulated by *Cyp26a1* encoded enzyme, which degrades excess RA [24, 25]. Thus, mutation of *Cyp26a1* gene results in the loss of its regulatory function of RA concentration within ECR milieu with consequent generation of SML phenotype [26, 27]. However, mutational sequencing of these putative genes have not been demonstrated in human fetuses with SML.

There are few cases of SML reported from Sub-Saharan Africa (SSA) [28]. To the best of our knowledge, the index case is the first to be reported from the East African region. Thus, the case serves to raise awareness among clinicians and highlights the importance pre-conception intervention as well as vigilant prenatal care (PNC) and surveillance.

## Case presentation

### Demographic details and medical history

A 22-year-old Black African woman, primigravida, married, a small-scale farmer with a primary level of education, and uneventful prepartum period presented to the hospital at full term in labor. She delivered a full-term fresh stillbirth by cesarean section due to obstructed labor with fetal distress. The mother booked prenatal clinic at a gestation age of 12 weeks and made only two visits throughout her prenatal period. During prenatal visit she was prescribed with routine supplements including anti-helminthes and sulfadoxine-pyrimethamine (SP) for presumptive treatment of malaria as well as ferrous sulfate and folic acid, which were not refilled afterward. She was reported as having normal blood pressure and blood glucose level, and tested negative for syphilis and human immunodeficiency virus (HIV) infection. However, she was not screened for other congenital fetomaternal infections, that is, TORCH complex. She had no history of active or passive smoking, alcohol consumption, drug abuse or any known familial congenital disorders. She denied any exposure to pesticides or herbicides, though reported regular use of fertilizers in her farming activities. She denied the use of post-coital hormonal contraceptives or misoprostol for attempted abortion of the index pregnancy. The parents of the newborn were not close relatives and there was no family history of consanguinity.

### Clinical findings

**Fig. 1:**
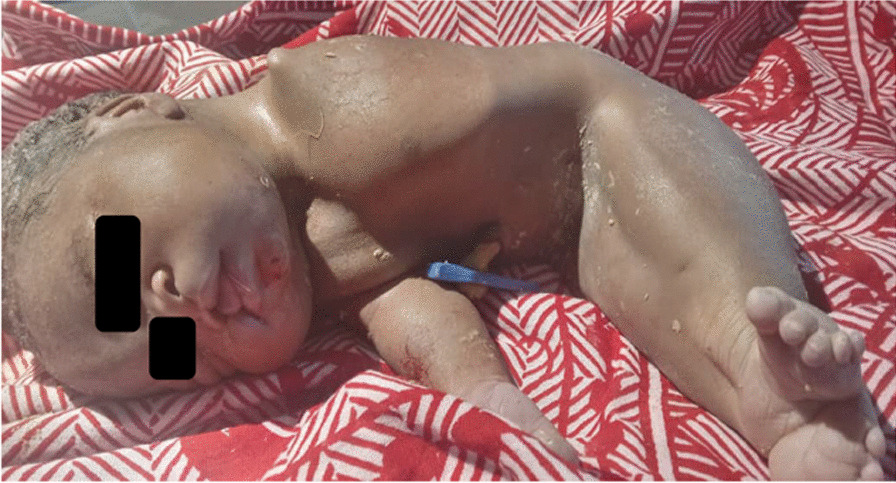
The newborn with sirenomelia showing visible fused lower limbs and absent one upper limb along with ambiguous genitalia

**Fig. 2:**
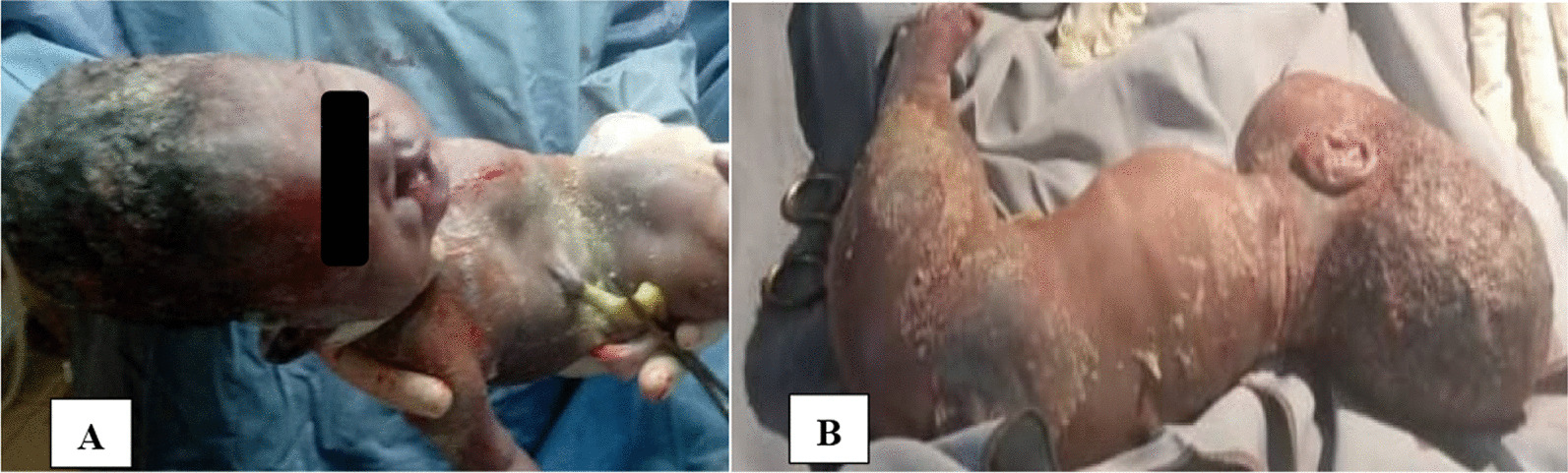
The newborn with sirenomelia: **A** anterior view showing a cleft lip; **B** posterolateral view showing absent right upper limb

The newborn was delivered by cesarean section in a standard manner. The newborn had an Apgar score of 0 both at 0 minutes and 5 minutes, consistent with a fresh stillbirth. On physical examination, the newborn weighed 2100 g, and had fused lower limbs, agenesis of the left upper limb, imperforate anus, ambiguous genitalia, and cleft lip as shown in Figs. 1 and 2. The mother did not have any peripartum complications.

### Diagnostic assessment

No any additional tests or imaging were performed. The diagnosis of SML was made on the basis of typical clinical features as depicted in Figs. 1 and 2. The differential diagnosis of CRS, Potter syndrome and a syndrome of vertebral (V), anorectal (A), cardiac (C), tracheoesophageal (TE), renal (R) and limb (L) anomalies - termed VACTERL associations, were less likely in this patient due to the obvious features suggestive of SML. Whole body imaging using x-ray and post mortem examination were not permitted due to cultural taboo and restriction regarding handling of deceased individuals. Further genetic testing was performed due to lack of laboratory capacity.

## Discussion

SML is a rare congenital anomaly that is invariably incompatible with extrauterine life due to multiple defects in critical systems [16, 29]. Thus, it could be speculated that most pregnancies of fetuses with SML could likely end up with abortions, contributing to the rarity of the condition postnatally. The syndrome is very rare and perhaps many healthcare professionals might not have come across a case of SML in their entire practice.

The pathogenesis and etiology of SML is poorly understood. However, it is believed to occur in individuals with a genetic predisposition involving genes responsible for ECR development, which is unmasked by poorly understood environmental triggers [16, 17, 23, 27]. In this index patient there was no identifiable risk factor for developing SML. However, the mother had very limited prenatal visits, which may have not provided enough time for pregnancy assessment. She was 21 years of age at the time of conception, which is not entirely different from the established age risk category from the literature, which is age below 20 years or above 40 years [4, 16]. Moreover, well-established infections such as TORCH complex, which have proven teratogenic potential, were not screened during PNC visit except for syphilis infection. Thus, the role of these infections could not be ruled out as the possible agents in the pathogenesis of SML. Studies from SSA and other developing countries indicate that the majority of pregnant women without regular PNC attendance are likely to seek healthcare attention from traditional healers, often without their disclosure to healthcare providers [30]. In this setting, women are potentially exposed to herbal concoctions without evident safety profile [30]. In addition, pregnant women without proper PNC care often miss the opportunity for routine supplementations with specific agents that have established value in the prevention of specific congenital anomalies [31].

SML is almost always fatal, thus, a high index of suspicious is required if early obstetric ultrasound is performed, with additional x-ray imaging or magnetic resonance imaging (MRI) in case of uncertainty [32, 33]. Early detection of SML provides an opportunity for counseling for the couple for possible termination of pregnancy [34, 35]. SML is invariably lethal within 1–2 days of birth because of the associated visceral malformation, however, survival could be prolonged through multidisciplinary surgical interventions [4, 7]. The index patient was a stillbirth, possibly highlighting the possible associated visceral malformations that were incompatible with perinatal life [7, 16].

This case report aimed to raise awareness for healthcare practitioners on the existence of this rare congenital anomaly. Moreover, we aimed to emphasize the importance of comprehensive reproductive health education spanning from preconception to the period throughout PNC. Furthermore, we emphasize incorporation of routine obstetric ultrasound imaging during the first trimester in the minimum PNC package for all women in SSA [35].

## Conclusion

SML is an extremely rare and fatal congenital anomaly with poor prognosis. Early obstetric ultrasound is critical for early detection of this syndrome. The termination of pregnancy is a recommended option if the diagnosis of SML is confirmed. However, potential for prevention should be sought as the goal through planned pre-conception strategies and comprehensive PNC package.

## Data Availability

The raw data pertaining to this case report are available on reasonable request.
